# Optimization of 3D Point Clouds of Oilseed Rape Plants Based on Time-of-Flight Cameras

**DOI:** 10.3390/s21020664

**Published:** 2021-01-19

**Authors:** Zhihong Ma, Dawei Sun, Haixia Xu, Yueming Zhu, Yong He, Haiyan Cen

**Affiliations:** 1 College of Biosystems Engineering and Food Science, Zhejiang University, Hangzhou 310058, China; zhma_20@zju.edu.cn (Z.M.); DZS0015@zju.edu.cn (D.S.); haixiaxu@zju.edu.cn (H.X.); zhukvo@zju.edu.cn (Y.Z.); yhe@zju.edu.cn (Y.H.); 2 Key Laboratory of Spectroscopy Sensing, Ministry of Agriculture and Rural Affairs, Hangzhou 310058, China; 3 State Key Laboratory of Modern Optical Instrumentation, Zhejiang University, Hangzhou 310027, China

**Keywords:** plants, 3D point cloud, Kinect, MOBB, mesh patches, optimization

## Abstract

Three-dimensional (3D) structure is an important morphological trait of plants for describing their growth and biotic/abiotic stress responses. Various methods have been developed for obtaining 3D plant data, but the data quality and equipment costs are the main factors limiting their development. Here, we propose a method to improve the quality of 3D plant data using the time-of-flight (TOF) camera Kinect V2. A K-dimension (k-d) tree was applied to spatial topological relationships for searching points. Background noise points were then removed with a minimum oriented bounding box (MOBB) with a pass-through filter, while outliers and flying pixel points were removed based on viewpoints and surface normals. After being smoothed with the bilateral filter, the 3D plant data were registered and meshed. We adjusted the mesh patches to eliminate layered points. The results showed that the patches were closer. The average distance between the patches was 1.88 × 10^−3^ m, and the average angle was 17.64°, which were 54.97% and 48.33% of those values before optimization. The proposed method performed better in reducing noise and the local layered-points phenomenon, and it could help to more accurately determine 3D structure parameters from point clouds and mesh models.

## 1. Introduction

With the increasing demand for accelerating plant breeding and improving crop-management efficiency, it is necessary to measure various phenotypic traits of plants in a high-throughput and accurate manner [1]. The fast development of advanced sensors and automation and computation tools further promotes the capability and throughput of plant-phenotyping techniques, which allows the nondestructive measurement of complex plant parameters or traits [2]. Plant three-dimensional (3D) morphological structure is an important descriptive trait of plant growth and development, as well as biotic/abiotic stress responses [3]. 3D plant phenotyping has great potential for multiscale analyses of the 3D morphological structures of plant organs, individuals and canopies; for building functional structure plant models (FSPM) [4], for evaluating the performance of different genotypes in adaptation to the environment, for predicting yield potential [5] and for facilitating the accurate management of breeding and crop production with key technical support.

Different 3D sensors and imaging techniques have been developed to quantify plants’ 3D morphological structural parameters at different scales. These sensors can be classified into passive and active sensors [6]. Generally, passive sensors build a 3D model from the images of different views. Some systems have been developed for obtaining a 3D model, such as an RGB camera combined with a structure from motion (SFM) algorithm and a multiview stereo vision system [7,8]. Rose et al. [9] found that the SFM-based photogrammetric method can yield high correlations to the measurements and was suitable for the task of organ-level plant phenotyping. Xiang et al. [10] developed a PhenoStereo system for field-based plant phenotyping and used a set of customized strobe lights for lighting influence. Rossi et al. [11] provided references for optimizing the reconstruction process of SFM in terms of input and time requirements. They found the proper balance between number of images and their quality for an efficient and accurate measurement of individual structural parameters for species with different canopy structures. However, methods combined with passive sensors have high requirements for images with complex features in the surface texture for image matching [6], and the methods are limited by lighting condition as well as the complexity of algorithm.

Active sensors acquire distance information from the active emission of signals [12]. Laser scanning is considered to be a universal, high-precision and wide-scale detection method for plant-growth status [5]. Paulus et al. [13] conducted a growth analysis experiment on eight pots of spring barley plants under different drought conditions in an industrial environment. Single leaf area, single stem height, plant height and plant width were determined with a laser-scanning system combined with an articulated measuring arm. These measurements had high correlations (R^2^, 0.85–0.97) with manual measurements. Based on such accuracy, they were also able to effectively monitor the growth and quantify the growth processes of barley plants. However, the small scanning field and small arm size necessitated multiple-location scans for whole plants, which made the system expensive and inefficient. Sun et al. [14] developed a system consisting of a 2D light detection and ranging (LiDAR) and real time kinematic global positioning system (RTK-GPS) for high-throughput phenotyping. They built a model to obtain the height of cotton plants, considering the angular resolution, sensor mounting height, tractor speed and so on. This system performed well in estimating the heights of cotton plants. However, many factors such as the angular resolution and uneven ground affected the measurements, and the data were noisy, which made it impossible to accurately measure other parameters such as the leaf area. Su et al. [15] proposed a difference-of-normals (DoN) method to separate corn leaves and stalks based on laser point clouds in a greenhouse. However, it took 20 min for each scan per position. Ana et al. [16] proposed a vine-shaped artificial object (VSAO) calibration method, based on which they implemented a static terrestrial laser scanner (TLS) and a mobile scanning system (MMS) with six algorithms to determine the trunk volumes of vines in a real vineyard. The results showed that the relative errors of the different sensors, combined with different algorithms, were 2.46%–6.40%. The limitations of these two systems included long scanning time, tedious processing and environmental factors. Laser scanner had high detection accuracy for individual plants and groups in industrial or field environments, but many factors such as the topography still impacted the measurements [17]. However, cost and the efficiency were the mainly bottlenecks that restricted the application of this technology in actual production.

Some other detection methods have been proposed, and laser scanning has become a common means of evaluating these methods [18,19,20]. Compared to laser scanning, the time-of-flight (TOF) camera has the advantages of speed, simplicity and low cost and the potential for use in 3D phenotyping research [20,21,22,23,24,25,26]. For example, Microsoft Kinect is widely used as a typical TOF camera. Paulus et al. [19] proved that a low-cost system based on the Microsoft Kinect device can effectively estimate the phenotypes of sugar beets. They used the David laser scanner system as a reference method. The results showed that the Kinect performed as well as the laser scanner for sugar-beet taproots in terms of height, width, volume and surface area estimation. However, the Kinect performed poorly in estimating wheat-ear parameters, due to the low resolution, while the laser scanner still performed well. The R^2^s of the maximum length and alpha shape volume were 0.02 and 0.40, respectively, when using Kinect, and the R^2^s of these two parameters were greater than 0.84 for the laser scan. Sugar beet is simple in morphology and structure; the potential of Kinect for other plants remains to be seen. Xia et al. [27] used a mean-shift-clustering algorithm to segment the leaves in depth images obtained from Kinect, and removed the background in both RGB and depth images. Based on the adjacent-pixel-gradient-vector-field of depth image, they achieved segmentation of shade leaves. This approach can be effectively applied to automatic fruit harvest and other agricultural automation work. However, their work only focused on a single-frame point cloud, which led to incomplete data for the plant. Meanwhile, the complete plant point clouds were more complex, with more noise and a layered-points phenomenon, which their algorithm could not solve. Anduja et al. [28] proposed reconstructing maize in the field with the Kinect Fusion algorithm. They monitored segmentation of maize, weeds and soil through height and RGB information and studied the correlation between volume and biomass. The coefficient of the correlation between the maize biomass and volume was 0.77, while that between the weed volume and biomass was 0.83. It was clear that the correlation coefficient was not as high as that in Paulus’s study [19] because of rough point clouds with poor quality caused by the complex field environment and complexity of the plants, and they did not perform point-cloud optimization. Wang et al. [29] measured the height of sorghum in the field using five different sensors and established digital elevation models. All the coefficients of correlation between the values generated by the models and those measured manually were above 0.9. They proposed that the Kinect could provide color and morphology information about plants for identification and counting. However, the data acquired by Kinect were, again, rough and noisy, and they were not suitable for the extraction of other parameters.

According to the above studies, multiple complex parameters were effectively extracted using laser scans because of the high-quality point clouds. Kinect, by contrast, performed well in height estimation and object segmentation because these two tasks do not require high-quality data. To extract more parameters efficiently in a low-cost platform, it was necessary to obtain complete and high-quality plant 3D data using a TOF camera. However, there was a layered-points phenomenon in the plant point clouds based on multiple frames [30] because of the errors from the TOF camera and registration algorithm, a common problem.

To improve the quality of the plant point cloud, we proposed an optimization method to reduce the impact of noise and layered-points. A simple and low-cost platform based on Kinect was used for data acquisition, which makes the proposed method more widely applicable. In this study, we optimized the quality of single-frame point clouds by removing all types of noise while preserving the integrity of the plant data. We also eliminated the local layered-points phenomenon to improve the quality of plant point clouds registered from multiple frames.

## 2. Materials and Methods

### 2.1. Experimental Setup and Data Acquisition

**Figure 1:**
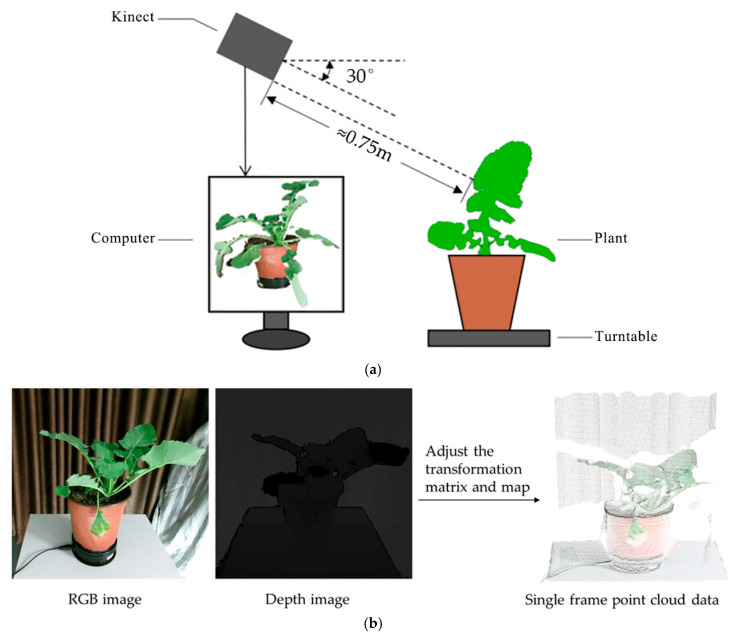
Acquisition system and point cloud acquisition. (**a**) Acquisition system; (**b**) the process of obtaining a single-frame point cloud.

The data used in this study were collected for one oilseed rape cultivar (*Brassica napus* L. cv. Zhe Da 619) in a closed indoor imaging platform, mainly comprising a Kinect V2 sensor, turntable and computer. The Kinect V2 (Windows version, Microsoft, Redmond, WA, USA) consisted of an RGB camera (1920 × 1080), near-infrared camera (512 × 424), and near-infrared light for acquiring color and depth data, respectively. The acquisition platform and point cloud acquisition are shown in Figure 1. As shown in Figure 1a, the Kinect V2 was about 0.75 m away from the main stem (vertical axis) of the plant, and the shooting angle was 30°. The rotary speed of the turntable was 14.4°/s for changing the plant pose, and the measured plant was placed on the center of the turntable. The computer controlled the Kinect V2 and acquired and processed the raw data. It had an Intel core i5-4590 processor, a Windows 10 64-bit operating system and 8GB of ECC RAM. Data processing was performed on Point Cloud Library (PCL) and Open3D Library in Visual studio 2013 (Professional version, Microsoft, Redmond, WA, USA). Before acquisition, the Kinect V2 camera was calibrated by Zhang’s [31] method, and the transformation matrix between the RGB and depth cameras was adjusted to optimize the mapping relationship for the two types of images to ensure the consistency of the color and depth of each point (Figure 1b).

**Figure 2:**
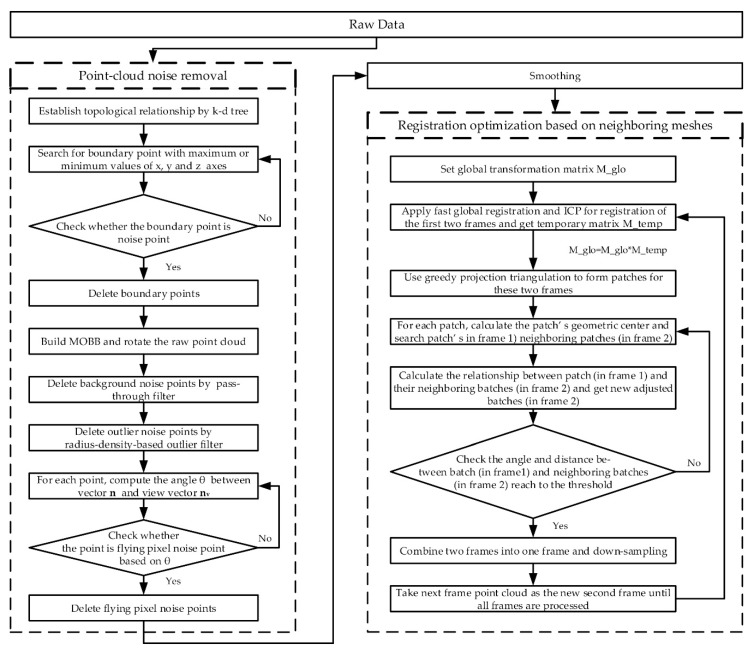
The flow chart of the proposed method.

A point-cloud-processing pipeline was developed to optimize the quality of the entire plant point cloud. As show in Figure 2, the pipeline of workflow mainly comprised three steps: (1) point-cloud noise removal; (2) point-cloud smoothing; (3) registration optimization based on neighboring meshes.

#### 2.1.1. Point-Cloud Noise Removal

The point cloud acquired by Kinect V2 was generally disordered, with many noise points that would have a significant effect on the reconstruction accuracy and computation speed. The viewpoint feature and normal feature of the point cloud were used to remove the noise based on the spatial topological relationship established by the k-dimensional (k-d) tree. The spatial topological relationship was used for searching neighboring points.

There were three types of noise in the point cloud: the background noise (BN), which consisted of nontarget points away from the targets; the outlier noise (ON), which consisted of scattered points, mostly around the targets, caused by the sensors, and the flying pixel noise (FPN) from the boundaries of two objects [32]. Traditionally, the BN has mainly been eliminated with a pass-through filter, and the ON removed based on the neighboring points. The pass-through filter limited the ranges of the X, Y and Z axes and removed the points outside the ranges. Due to FPN, points covering different objects of different depths were distant. The vector made up of FPN and viewer points was almost perpendicular to the FPN point’s normal vector. The FPN points could be removed based on these two features.

**Figure 3:**
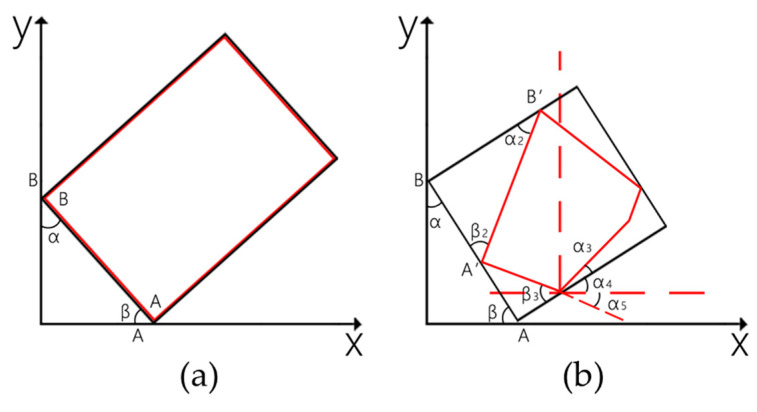
The relationship between the object, minimum oriented bounding box (MOBB) and coordinate in 2D space, (**a**) under ideal conditions and (**b**) under general conditions.

Because the central axis of the plant is not strictly perpendicular to the camera-projection direction during data acquisition, it is very difficult to eliminate the BN points while preserving the integrity of the plant by using the traditional pass-through filter method. In this study, a combination of a pass-through filter and minimum oriented bounding box (MOBB) was proposed. The MOBB was a cuboid that contained the object as tightly as possible, with the smallest volume in the defined coordinate system. In 2D space, assuming the camera tilt angle was θ, this coordinate was aligned parallel to the camera coordinate. If the data of the object (red box) had a rectangular distribution under the ideal condition as shown in Figure 3a, the MOBB (black box) was equivalent to this rectangle. In this case, after the MOBB was rotated in the β counterclockwise direction around Point A, the object was aligned and parallel to the camera coordinate, and θ=β. Normally, the distribution of the object was irregular (red box) as shown in Figure 3b. The relationship between the angles was calculated as below:

(1) $$\theta =\frac{\pi }{2}-\alpha_{3},$$

(2) $$\beta_{2}\;=\beta_{3}=\alpha_{2}+\alpha_{3};$$

(3) $$\alpha_{2}\;=\alpha ;$$

(4) $$\beta_{2}=\frac{\pi }{2}-\theta +\alpha ;$$

where *θ* is the camera tilt angle, α, $\alpha_{2}$ and β are the angles between the MOBB and camera coordinate and $\alpha_{3}$ is the angle between the object box and camera coordinate. $\beta_{2}$ and $\beta_{3}$ are the angles between the MOBB and object box and were equivalent in value.

In this case, α and β can be obtained from the MOBB orthogonal and the camera coordinates. After the object was rotated in $\beta_{2}$ counterclockwise around Point A’, firstly, and the MOBB was rotated β counterclockwise around Point A, secondly, the object was aligned and parallel to the camera coordinate. The same method was applied to 3D space, in which the rotation of the object was achieved with Euler’s formula. The MOBB orthogonal coordinate was established with the center of the point cloud data as the coordinate origin and the length, width and height of the MOBB, which can improve the performance of the pass-through filter.

After the removal of the BN points, there were still many ON and FPN points that needed to be removed. A radius-density-based outlier filter was implemented to remove the ON points [33]. For each point $p^{i}$ of data, it takes into account both the number K and the average distance $\bar{d}\left(p\right)$ of the neighboring points within a certain radius *r* of the selected point. If the selected point was judged as ON, the following conditions were met:

(5) $$\bar{d}\left(p\right)=\frac{1}{K}\sum_{p_{j}\in N\left(p\right)}\|p-p_{j}\|$$

(6) $$\bar{d}\left(p\right)>\left(\mu +n\cdot \sigma \right)$$

(7) K>k

where $p_{j}$ is the neighboring point of the selected point *p*, μ is the average distance between neighboring points, σ is the standard deviation of μ, *n* is the multiple of σ, and k is the defined point number.

As for FPN, it can be removed based on the angle θ of the normal vector $\vec{n_{i}}$ and the view vector $\vec{n_{v}}$ connecting with the viewpoint. The viewer vector consists of the viewpoint $\vec{n_{v}}$ and p. For each point p, if θ was bigger than the threshold $\theta_{angle}$, this point could be removed as FPN. The noise-elimination process is summarized in Algorithm 1 as shown below.
**Algorithm 1:** Point-cloud-noise removalInput: Raw data of point cloud $\left\{p_{in}\right\}$
Output: Object point cloud $\left\{p_{filter-out}\right\}$ without noise.(1) Establishing the spatial topological relationship of the source data using the k-d tree.(2) Obtaining the maximum and minimum values of the three coordinate axes in the point cloud and searching six boundary points with $x_{min}$, $y_{min}$, $z_{min}$, $x_{max}$, $y_{max}$ and $z_{max}$ respectively. A radius-density-based outlier filter is used on these six points. If they are outliers, then delete and repeat this step. Otherwise, proceed to the next step.(3) Building up the MOBB, rotating it with Euler’s formula and removing BN points using a pass-through filter.(4) Removing ON points using the radius-density-based outlier filter for all points.(5) For each point of data, computing the normal vector $\vec{n}$ of the selected point p by principal component analysis (PCA). Computing the components of the normal vector $\vec{n}$ and the view vector $\vec{n_{v}}$ projection on the xoz plane of the camera coordinate space separately, and then obtaining angle θ through the cosine theorem.(6) Comparing θ and $\theta_{angle}$ and removing FPN points.(7) Performing Operations 4 through 7 on all points and outputting point $\left\{p_{filter-out}\right\}$.

In order to evaluate the effect of noise removal, the benchmark point cloud was segmented manually in Geomagic Studio [34]. Thus, the valid-point percent (VPP) was proposed. The closer the VPP to 100%, the fewer the non-target points.

(8) VPP=Valid points/Total points

#### 2.1.2. Point-Cloud Smoothing

The bilateral filter is a nonlinear filtration tool used for edge-preserving smoothing [35]. Due to the wiggling error caused by the Kinect sensor, the fitting surface of the data acquired by the Kinect was not smooth [22]; this could be solved by this filter. Several 3D bilateral filters are based on the mesh model [36,37]. However, the mesh or fitting surface was easily affected by the noise. Based on the neighboring points, the disordered bilateral filter was used to smooth the point cloud while preserving the edge features of the point cloud [33].

(9) $$\alpha =\frac{\sum_{p\in \left\{p^{i}{}_{r}\right\}}(W_{c}\left(\|p^{i}-p\|\right)W_{s}\left(\|\vec{p^{i}-p},\vec{n_{i}}\|\right)\left(\vec{p^{i}-p}\cdot \vec{n_{i}}\right))}{\sum_{p\in \left\{p^{i}{}_{r}\right\}}W_{c}\left(\|p^{i}-p\|\right)W_{s}\left(\|\vec{p^{i}-p},\vec{n_{i}}\|\right)}$$

(10) $$p^{\prime }=p^{i}+\alpha *\vec{n_{i}}$$

(11) $$W_{c}\left(\|p^{i}-p\|\right)=exp\left[-\frac{{\left\|p^{i}-p\right\|}^{2}}{2\sigma_{c}^{2}}\right]$$

(12) $$W_{s}\left(\|p^{i}-p\|\right)=exp\left[-\frac{{\left\|p^{i}-p\right\|}^{2}}{2\sigma_{s}^{2}}\right]$$

where $p^{i}$ is the selected point and $\left\{p^{i}{}_{r}\right\}$ are the neighboring points within the radius of r. $W_{c}$ is related to the smoothness and $\sigma_{c}$ is the distance factor. $W_{s}$ is related to the ability to preserve features and $\sigma_{s}$ is the hue factor.

#### 2.1.3. Registration Optimization Based on Neighboring Meshes

**Figure 4:**
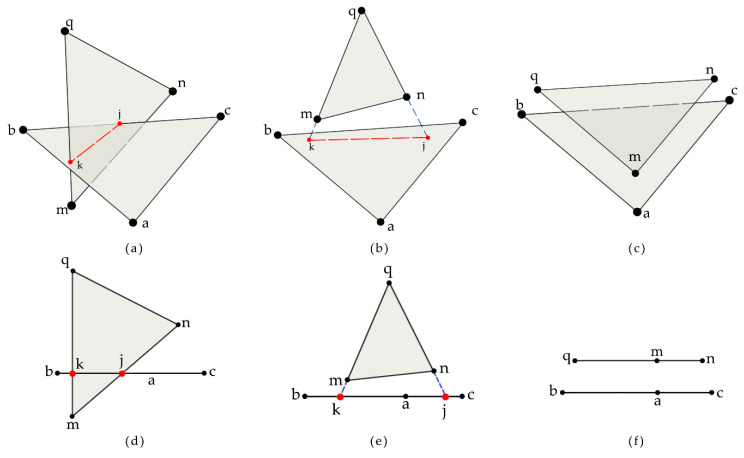
Relationship between triangular patches, (**a**–**c**) are 3D view images while (**d**–**f**) are front view images. (**a**,**d**) Intersecting patches. (**b**,**e**) Plane intersecting patches. (**c**,**f**) Parallel patches.

The purpose of registration was to unify the point clouds from different coordinate systems into the same coordinate system [38]. Multiple neighboring point clouds were registered into a single point cloud using fast-point-feature histograms (FPFH) for rough registration and an iterative-closest-point (ICP) algorithm for fine alignment [39]. However, the local layered points could be observed after registration due to the complex refraction and reflection situations in the interiors or surfaces of the leaves [30]. The accuracy of the algorithm also affected the layered-points phenomenon. These stratified leaves were close, and the layered-points phenomenon could be optimized by adjusting the related points’ position. In the point-cloud model, there was no geometrical relationship between the points, and the topological relation supported by the k-d tree was only applicable to searching neighboring points. A mesh model based on a triangular patch was more suitable for solving the issue of layered points. Three definitions were proposed to explain the triangular patch relationship in Figure 4. The symbol △ stands for a triangle patch.

**Definition** **1.** *Intersecting relationship: there are patches* △abc *and* △mnq *where at least one edge (including the vertices) of* △abc *intersects the plane where* △mnq *is located, and the intersection point is inside* △mnq *and also on the edge of* △abc *.*

**Definition** **2.** *Plane intersecting relationship: there are patches* △abc *and* △mnq *, where the plane in which* △abc *lies intersects the plane in which* △mnq *lies, and the intersection point is inside* △mnq *and also on the extension line of the edge of* △abc *.*

**Definition** **3.** *Parallel relationship: there are patches* △abc *and* △mnq *, and the plane in which* △abc *lies is parallel to the plane in which* △mnq *lies.*

Figure 4a–c shows 3D view images while Figure 4d–f shows front view images. We assume that the △abc is parallel to the horizontal plane, so it looks like a line in the front view images (Figure 4a–c). According to Figure 4a,d, the △abc and △mnq have an intersecting relationship. The red points k and j are the intersection points of these two patches, and the red dotted line k-j is the intersecting line. The intersecting points and intersecting line are all inside two patches. According to Figure 4b,e, the △abc and △mnq have a plane intersecting relationship. It means the plane in which △abc lies intersects the plane in which △mnq lies. However, the intersecting points and intersecting line are only inside the △abc while outside the △mnq, and intersecting points k and j are in the extension lines (blue dotted lines) of m-q and n-q respectively. According to Figure 4c,f, the △abc and △mnq have a parallel relationship and the △abc is parallel to the △mnq.

Based on these three definitions, two frames of the point cloud used for registration were meshed by using the greedy-projection-triangulation algorithm. Supposing that △abc was one patch of the first frame point cloud, △mnq was the neighboring patch of △abc in the second frame and $p_{mid}$ was the median plane of these two patches, the angle $\alpha_{tri}$ and distance $d_{tri}$ were then calculated. If the $\sin^{-1}\alpha_{tri}$ was less than $10^{-6}$, these two patches were parallel, otherwise, the relation of these two patches needed to be computed using Möller’s theory [40]. In the intersecting or plane intersecting relationships, if $\alpha_{tri}$ was larger than $\alpha_{tri-min}$, each vertex of △mnq was projected onto the $p_{mid}$ forming a new patch △m′n′q′. However, in the plane intersecting relationship, the distance $d_{pro}$ between the projection point and origin point was considered. If $d_{pro}$ was bigger than the point moving threshold $d_{pro-max}$, which meant that these two patches were not close enough, the projection operation was cancelled. Meanwhile, for the parallel condition, both △abc and △mnq were projected onto the $p_{mid}$, forming two new patches, △a′b′c′ and △m′n′q′. After projection, the distance $d_{cen}$ between the geometric centers of two new patches was the basis for determining whether the projection operation was effective or not. If $d_{cen}$ was larger than the threshold $d_{cen-max}$, which meant that these two patches were not close enough, the projection operation was cancelled. However, the retrieval of the proposed neighboring patches was based on the k-d tree and patches’ geometric center, so $d_{pro}$ was always less than $d_{pro-max}$ and $d_{cen}$ was always less than $d_{cen-max}$. Iteration produced the best result for two frames, and incremental registration optimization made all the frames into one. The detailed optimization algorithm is presented in Algorithm 2.
**Algorithm 2:** Registration optimization based on neighboring meshesInput: different frames point clouds of plant after denoising and smoothingOutput: one frame of complete plant point cloudSetting: global transformation matrix $M_{glo}$
(1) Registration: At the beginning, the first two frames are selected for processing. Fast global registration [41], which is more efficient than FPFH, is applied for rough registration, and ICP is applied for fine alignment, producing the temporary matrix $M_{temp}$.
$$M_{glo}=M_{glo}*M_{temp}$$
(2) Meshing: Greedy projection triangulation is used to form triangular patches for these two frames.(3) Searching neighboring patches: Calculating the patches’ geometric center and getting two center point clouds $p_{c1}$ and $p_{c2}$. For each point in $p_{c1}$, searching the neighboring points of selected point in $p_{c2}$ based on the k-d tree. The center point corresponds to the patch, so the neighboring patches of the patch △abc of $p_{c1}$ are a set $T=\{△mnq_{i}\;|\;i=1,2,3\ldots \ldots \}$
(4) Calculating the relationship between the patches: For each patch in set T, calculating the relationship between this patch and patch △abc. After projection, the new patch will take the place of the old one.(5) Iteration and repetition: If $\alpha_{tri}$ is less than the minimum angle threshold $\alpha_{min}$, or $d_{pro}$ is less than the minimum distance threshold $d_{min}$, the optimization of these pairs of patches is completed. Repeating Steps 3–5 for all patches in the first frame. (6) Down-sampling: After optimization, these two frames are combined into one frame, which is set as the new first frame. Due to repeated points, down-sampling is applied to reduce the point-cloud density.(7) Applying to all frames: Taking the next frame from memory as the new second frame and then repeating the above operations until all frames are used.

## 3. Results and Discussion

The experiments were carried out on raw data obtained from 10 pots of oilseed rape. For each pot of plant, 10 frames of point cloud data from different views, which cover 360°and these data were processed by the proposed method to show the performance and robustness of proposed method.

### 3.1. Point-Cloud Noise Removal

**Figure 5:**
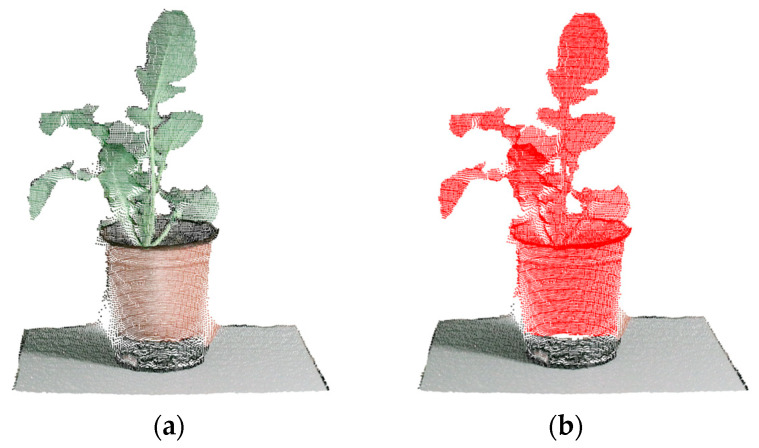
The valid points (red points). (**a**) Original point cloud. (**b**) Original point cloud with valid points.

**Figure 6:**
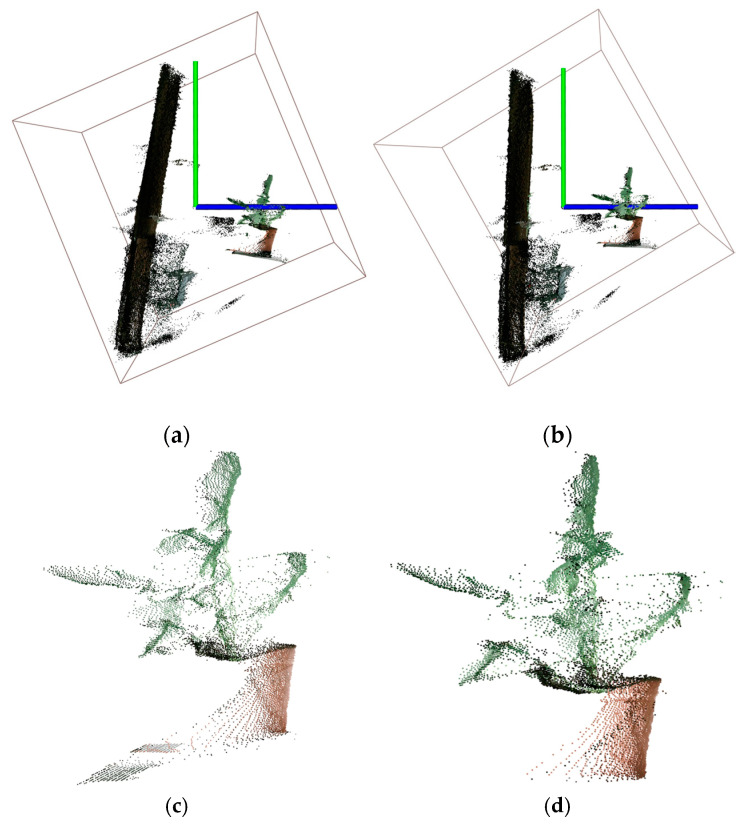
Removal of BN points. (**a**) Original point cloud. (**b**) Point cloud rotated by MOBB. (**c**) Original point cloud after filtering with pass-through filter. (**d**) The rotated point cloud after filtering with pass-through filter.

At the beginning, there were approximately 210,000 points of raw date in each frame. Most of them were noise points, as shown in Figure 5a and Figure 6a. According to the definition in Section 2.1.1 and function (8), the red points in Figure 5b were valid points, and other points were noise points. The performance of removing BN points was evaluated by VPP. However, the perpendicular requirement, mentioned in Section 2.1.1, between the center axis of the plant and the camera-projection direction was not strictly satisfied (Figure 6a), the data still retained lots of BN points after using the pass-through filter directly (Figure 6c). As shown in Figure 6b, the point cloud data was rotated by MOBB to satisfy the perpendicular requirement, then BN points were removed more effectively by pass-through filter (Figure 6d). The comparison results between the above two methods for removing BN points in 10 frames of point cloud of one plant (plant 1) are presented in Table 1. In this experiment, the thresholds of the pass-through filter were (−9,40), (−25,50) and (35,70) cm in the X, Y and Z directions, respectively. These thresholds can preserve a more complete plant point cloud. Compared with the average VPP of the pass-through filter, which was 75.64%, the average VPP of the pass-through filter based on the MOBB was 92.05%. Several valid points removed by the method based on MOBB were mostly FPN points and accounted for a small number, so the method wouldn’t affect the quality of the point cloud. Table 2 shows the results of 10 pots of plants applied with above methods. The average VPP (AVPP), which was the average value of 10 frames’ VPP of each plant, was used. The AVPP still remained at a high level, with an average value of 92.28% and standard deviation (SD) of 2.27. According to the VPP and AVPP, the performance and the robustness of the proposed method was revealed by higher average value and smaller SD value, which indicated that the proposed method performed well with different frames of point cloud of a plant and different plants.

**Figure 7:**
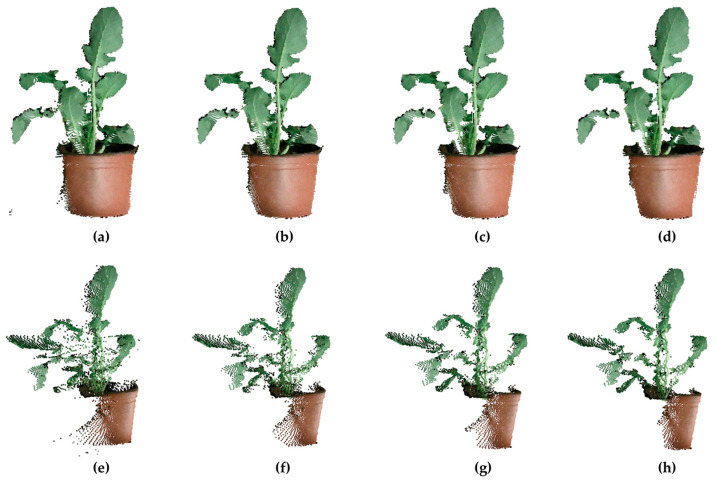
Results of different denoising methods: (**a**–**d**) front view images, (**e**–**h**) side view images. (**a**,**e**) The original point cloud. (**b**,**f**) The result after using the radius-based outlier filter. (**c**,**g**) The result after using the radius-density-based outlier filter. (**d**,**h**) The result after using the proposed method.

After removing BN points, there were still many ON and FPN points (Figure 7a,e). According to groups of experiments [33,42], the point cloud has good quality when r = 2 mm, K = 30, n = 2, and θ = 85°. As shown on the front view images in Figure 7a–d, all methods performed well in removing ON points. However, when it was switched to the side view, the results in Figure 7e–h indicated that there were significant differences between several methods. In Figure 7f, the data filtered by the radius-based outlier filter still contained many ON points around the leaves. As shown in Figure 7g,h, both the radius-density-based outlier filter and the proposed method generated relatively clean data. As mentioned in Section 2.1.1, FPN points existed at the edge of the leaf but were different from ON points, so the radius-density-based outlier filter could not deal with FPN points well. The proposed method got a better result by removing more FPN points on the boundary of the pot of the plant and ON points outside the leaves. As presented in Table 3, the radius-density-based outlier filter removed more ON points and had a higher average noise-reduction ratio (NRR) compared with the radius-based outlier filter. Further, considering the fact that the proposed method removed more FPN and ON points than the other two methods, it was reasonable that the proposed method reached average noise-reduction ratio of 14.06%. It was noteworthy that at the locations close to the boundary of leaves, the proposed method would mistake a few boundary points for FPN points and removed them from the point cloud, which brought a big SD of the noise-reduction ratio in Table 3. As for the whole plant, the proposed method showed comparable performance in removing ON and FPN points with high noise-reduction ratio (Table 4).

Above all, the proposed method performed well both in different frames of point cloud data of one plant and data of different plants. The small SDs from Table 1, Table 2, Table 3 and Table 4 indicated that the method had strong robustness.

### 3.2. Point-Cloud Smoothing

**Figure 8:**
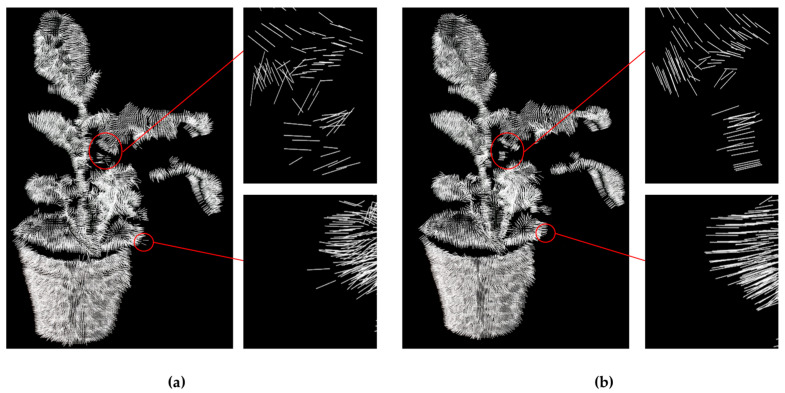
The distributions of the normals of (**a**) the original points and (**b**) the points after smoothing.

The smoothing effect of the bilateral filter mainly depends on $\sigma_{c}$ and $\sigma_{s}$. The larger $\sigma_{c}$, the smoother the point cloud was after processing. At the same time, the larger $\sigma_{s}$, the more the point-cloud features that were preserved after processing. The optimal $\sigma_{c}$ and $\sigma_{s}$ were determined based on the different datasets acquired in this study. As shown in Figure 8, when $\sigma_{c}$ = 10 and $\sigma_{s}$ = 0.1, the distribution of the normals of the points was neat, which meant that the point cloud was smooth.

### 3.3. Optimization of Registration Based on Neighboring Meshes

The method proposed in this study was based on neighboring meshes, so the triangulation algorithm had a certain influence on the processing results. Meanwhile, the number of neighboring meshes processed also affected the results. If there are too many meshes, overlapping may occur. According to several sets of experiments, the optimization effect was best when $\alpha_{min}$ = 20°, $d_{min}$ = 2 × the distance of the patches’ geometric center, the maximum number of iterations was 100, and the number of neighboring patches ≤ 3. Under this condition, 10 groups from different views covering 360° were tested, and each group had two adjacent frames of point cloud data. As shown in Table 5, the average Euclidean distance (AveEd) between parallel patches after optimization was 2.65 × 10^−3^ mm, and the average angle (AveAn) between intersecting and plane intersecting patches was 17.30°, which were 64.79% and 42.07% of these values before optimization, respectively. The smaller distance and angle indicated that the optimization method made neighboring patches from different frames of point cloud data become more appressed. The SDs of AveEd and AveAn after registration with optimization were low, which indicates that the optimization method had strong robustness. According to Table 6, the AveEd and AveAn were close to half of those value before optimization. The optimization method performed well in different plants stably with small SDs (Table 6).

**Figure 9:**
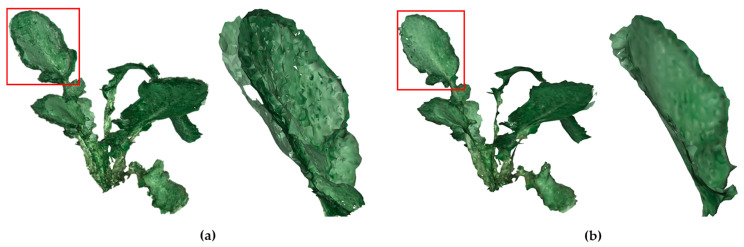
The results for the point cloud after registration: (**a**) Meshes of point cloud without optimization. (**b**) Meshes of point cloud after optimization.

From the above results, the proposed methods including the point-cloud noise removal method and the optimization method proved to have good performance and strong robustness not only in different frames of point cloud data of one plant but also different plants. Thus, we used 80 frames of data of one plant, which covered 360° to obtain a complete plant. 80 frames ensured a small angle between adjacent frames. Comparing Figure 9a with Figure 9b, the local layered points phenomenon improved. The leaf had three layers (in the red box of Figure 9a) before optimization, while it only had one layer (in the red box of Figure 9b) after optimization.

### 3.4. Efficiency

**Figure 10:**
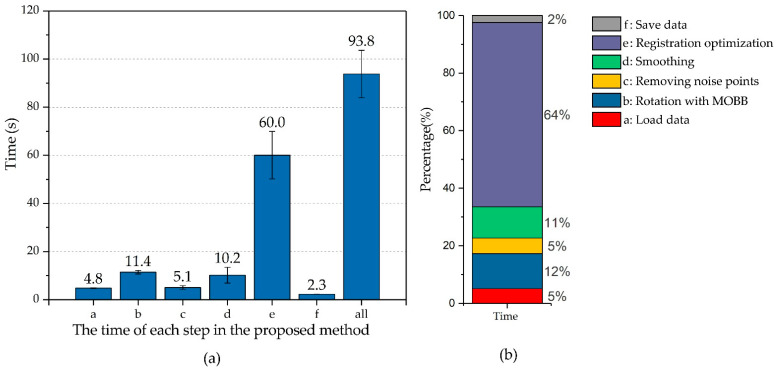
(**a**) The time of each step in the proposed method. (**b**) The proportion of the time cost for each step.

In order to obtain the point cloud data of a complete plant, we used 80 frames of point cloud data. In the tests of 10 pots of different plants, the average total time taken for the acquisition of the point cloud data of a complete plant was about 93.8 s, and the number of output plant points about one hundred thousand. Figure 10a illustrates the time consumed for each step in the proposed method. The longest time consumed by method was registration optimization based on neighboring meshes, which accounted for 64% of the total time consumed (Figure 10b). The calculation would be much faster if multi-thread processing was applied on a high configuration computer.

## 4. Conclusions

The plant 3D point-cloud optimization method proposed in this paper proved to be reliable for improving the quality of the plant point cloud. The point cloud was rotated into a better pose based on MOBB, and background noise points were totally removed with a pass-through filter, which preserved more valid points. For different plants, the method kept the valid point percent up to 92.28%, while that value was 82.24% only using pass-through filter. It was applicable to the plant-point-cloud data without plane objects due to the MOBB. The viewpoints and surface normals were effective in removing the outlier noise points and flying pixel noise points. In addition, we proposed applying neighboring mesh patches optimization during registration. After optimization, the average distance between the patches was 1.88 × 10^−3^ mm, and the average angle was 17.64°, which were 54.97% and 48.33% of those values before optimization, respectively. The impact of the layered-points phenomenon was effectively reduced, and the quality of the plant data were improved. The proposed method offers the potential to obtain complete and accurate plant data and may help to promote the popularization of plant-phenotyping research with low-cost sensors.

## Figures and Tables

**Table 1:** The comparison results of two methods for removing background noise points from one plant (plant 1).

Frame Number	Total Valid Points	Pass-Through Filter			Pass-Through Filter Based on MOBB		
		Valid Points	Total Points	VPP %	Valid Points	Total Points	VPP %
1	12,690	12,686	16,819	75.43	12,509	13,477	92.82
2	12,638	12,637	16,841	75.04	12,566	13,580	92.53
3	12,600	12,595	16,775	75.08	12427	13,613	91.29
4	12,669	12,650	16,780	75.39	12,502	13,660	91.52
5	12,526	12,506	16,801	74.44	12,436	13,622	91.29
6	12,807	12,785	16,925	75.54	12,659	13,764	91.97
7	12,837	12,803	16,854	75.96	12,773	13,788	92.64
8	12,893	12,859	16,902	76.08	12,726	13,826	92.04
9	12,911	12,878	16,895	76.22	12,689	13,812	91.87
10	13,096	13,033	16,880	77.21	12,869	13,904	92.56
Average	\	\	\	75.64	\	\	92.05
SD	\	\	\	0.73	\	\	0.54

Note: MOBB represents the minimum oriented bounding box. VPP represents the valid-point percent. SD represents the standard deviation.

**Table 2:** The average valid point percent of 10 pots of plants (%).

Method	Plant Number											
	1	2	3	4	5	6	7	8	9	10	Average	SD
**Method A**	75.64	84.31	87.97	77.30	84.03	83.19	82.95	80.40	82.95	82.57	82.24	3.37
**Method B**	92.05	89.67	94.28	97.10	92.80	94.29	89.37	90.50	91.68	91.08	92.28	2.27

Note: Method A is pass-through filter method, and method B is pass-through filter based on MOBB method. MOBB represents the minimum oriented bounding box. VPP represents the valid-point percent. SD represents the standard deviation.

**Table 3:** The results of three methods for removing outlier noise and flying pixel noise points from one plant (plant 1).

Frame Number	Original Points	Radius-Based Outlier Filter		Radius-Density-Based Outlier Filter		The Proposed Method	
		Points	NRR/%	Points	NRR/%	Points	NRR/%
1	13,477	13,385	0.68	12,792	5.08	11,107	17.59
2	13,580	13,481	0.73	12,792	5.8	11,691	13.91
3	13,613	13,516	0.71	12,779	6.13	11,737	13.78
4	13,660	13,534	0.92	12,772	6.5	11,722	14.19
5	13,622	13,488	0.98	12,792	6.09	11,697	14.35
6	13,764	13,629	0.98	12,962	5.83	11,789	14.67
7	13,788	13,685	0.75	12,935	6.19	11,765	14.7
8	13,826	13,611	1.56	12,929	6.49	11,793	17.41
9	13,812	13,630	1.32	12,941	6.31	11,407	14.62
10	13,904	13,698	1.48	12,975	6.68	11,871	14.13
Average	\	\	1.01	\	6.11	\	14.94
SD	\	\	0.33	\	0.46	\	1.39

Note: NRR represents Noise reduction ratio. NRR = (1 − point number after denoising/original point number) ∗ 100%. SD represents standard deviation.

**Table 4:** The noise reduction ratio (%) for 10 pots of plant from three methods.

Method	Plant Number											
	1	2	3	4	5	6	7	8	9	10	Average	SD
Method A	1.01	1.27	1.82	2.49	2.53	0.58	1.05	1.97	3.46	1.82	1.80	0.87
Method B	6.11	7.02	7.12	8.08	7.91	4.97	6.80	7.21	8.11	7.19	7.05	0.96
Method C	14.94	9.95	10.15	10.86	12.54	9.78	10.78	11.16	10.98	11.25	11.24	1.52

Note: Method C is radius-based outlier filter method, method B is radius-density-based outlier filter MOBB method and method C is the proposed method. SD represents the standard deviation.

**Table 5:** The effects of registration optimization of one plant (plant 1).

Test Group	Before Registration		After Registration without Optimization		After Registration with Optimization	
	AveEd $/10^{-3}\mathrm{mm}$	AveAn/°	AveEd $/10^{-3}\mathrm{mm}$	AveAn/°	AveEd $/10^{-3}\mathrm{mm}$	AveAn/°
1	3.96	41.46	4.20	41.09	3.56	16.80
2	3.94	41.46	4.33	41.25	2.85	17.19
3	3.77	41.16	4.41	40.72	2.82	16.66
4	3.86	41.49	4.11	40.89	3.07	16.85
5	4.24	41.04	4.63	40.73	2.93	17.32
6	3.92	41.57	4.07	40.91	3.01	17.37
7	3.42	41.99	3.71	41.04	2.84	17.38
8	3.74	41.86	3.99	41.81	1.60	17.74
9	3.19	41.69	3.72	41.34	1.38	18.09
10	3.23	41.43	3.77	41.47	2.45	17.58
Average	3.73	41.52	4.09	41.12	2.65	17.30
SD	0.34	0.29	0.31	0.35	0.67	0.44

Note: AveEd represents the average Euclidean distance of parallel triangles in the neighborhood, and AveAn represents the average angle of cross triangles or intersecting triangles in the neighborhood. SD represents standard deviation.

**Table 6:** The effects of registration optimization of 10 pots of plant.

The Evaluation Index	Plant Number											
	1	2	3	4	5	6	7	8	9	10	Average	SD
A	41.52	37	37.07	37.23	37.34	37.56	38.31	37.95	37.78	37.98	37.97	1.32
B	3.73	4.82	4.4	3.41	6.56	3.96	4.35	3.42	3.67	5.33	4.37	0.99
C	41.12	35.15	35.99	35.46	35.8	36.54	36.67	36.08	36.2	36.01	36.50	1.68
D	4.09	3.92	2.36	2.7	3.8	3.08	3.62	3.58	3.6	3.42	3.42	0.55
E	17.3	17.7	17.22	17.57	18.12	18.32	17.44	17.64	17.42	17.64	17.64	0.35
F	2.65	2.58	1.59	1.1	1.58	1.73	2.23	1.74	1.75	1.81	1.88	0.48
E/C (%)	42.07	50.36	47.85	49.55	50.61	50.14	47.56	48.89	48.12	48.99	48.33	2.47
F/D (%)	64.79	65.82	67.37	40.74	41.58	56.17	61.60	48.60	48.61	52.92	54.97	9.89

Note: A is the AveEd ($10^{-3}$ mm) before registration; B is the AveAn (°) before registration; C is the AveEd ($10^{-3}$ mm) after registration without optimization; D is the AveAn (°) after registration without optimization; E is the AveEd ($10^{-3}$ mm) after registration with optimization; F is the AveAn (°) after registration with optimization. SD represents standard deviation.

## Data Availability

Data sharing not applicable.
